# Microglial deletion and inhibition alleviate behavior of post-traumatic stress disorder in mice

**DOI:** 10.1186/s12974-020-02069-9

**Published:** 2021-01-05

**Authors:** Shuoshuo Li, Yajin Liao, Yuan Dong, Xiaoheng Li, Jun Li, Yong Cheng, Jinbo Cheng, Zengqiang Yuan

**Affiliations:** 1 The Brain Science Center, Beijing Institute of Basic Medical Sciences, No. 27 Taiping Road, Haidian District, Beijing, 100850 China; 2 Center on Translational Neuroscience, College of Life & Environmental Science, Minzu University of China, Beijing, 100081 China; 3 Department of Biochemistry, Medical College, Qingdao University, Qingdao, 266071 Shandong China; 4 Beijing Institute for Brain Disorders, Capital Medical University, Beijing, 100069 China

**Keywords:** Mass cytometry, Microglia, Microglial depletion, Microglial activation, PTSD

## Abstract

**Background:**

Alteration of immune status in the central nervous system (CNS) has been implicated in the development of post-traumatic stress disorder (PTSD). However, the nature of overall changes in brain immunocyte landscape in PTSD condition remains unclear.

**Methods:**

We constructed a mouse PTSD model by electric foot-shocks followed by contextual reminders and verified the PTSD-related symptoms by behavior test (including contextual freezing test, open-field test, and elevated plus maze test). We examined the immunocyte panorama in the brains of the naïve or PTSD mice by using single-cell mass cytometry. Microglia number and morphological changes in the hippocampus, prefrontal cortex, and amygdala were analyzed by histopathological methods. The gene expression changes of those microglia were detected by quantitative real-time PCR. Genetic/pharmacological depletion of microglia or minocycline treatment before foot-shocks exposure was performed to study the role of microglia in PTSD development and progress.

**Results:**

We found microglia are the major brain immune cells that respond to PTSD. The number of microglia and ratio of microglia to immunocytes was significantly increased on the fifth day of foot-shock exposure. Furthermore, morphological analysis and gene expression profiling revealed temporal patterns of microglial activation in the hippocampus of the PTSD brains. Importantly, we found that genetic/pharmacological depletion of microglia or minocycline treatment before foot-shock exposure alleviated PTSD-associated anxiety and contextual fear.

**Conclusion:**

Our results demonstrated a critical role for microglial activation in PTSD development and a potential therapeutic strategy for the clinical treatment of PTSD in the form of microglial inhibition.

**Supplementary Information:**

The online version contains supplementary material available at 10.1186/s12974-020-02069-9.

## Introduction

Post-traumatic stress disorder (PTSD) is a psychiatric disorder that develops after an individual is exposed to traumatic events. Although a commonly occurring disorder, the mechanisms underlying PTSD development remain unclear. A growing body of evidence shows that imbalances in the immune system and hyperactive neuroinflammatory responses may play a role in the development of PTSD [1, 2]. In the peripheral immune system, PTSD patients have shown higher numbers of Th1 cells and impaired function of Treg cells [3, 4]. Moreover, some pro-inflammatory cytokines including tumor necrosis factor α (TNFα), interleukin-6 (IL-6), and interleukin-1β (IL-1β) were also significantly increased in the serum of PTSD patients [5, 6]. Therefore, neuroinflammation might be a significant contributor to PTSD pathology.

Microglia constitute the major type of immune cells in the central nervous system (CNS) and are involved not only in the development of Alzheimer’s disease [7], Parkinson’s disease [8], and stroke [9], but also in depression by regulating synaptic function [10]. Recently, RNA sequencing results from PTSD model mice showed differential expression patterns of cytokines in various areas of the brain [11]. Microglia-derived pro-inflammatory cytokines are correlated with affective behaviors [12], and an endotoxin challenge has been shown to activate microglia, leading to negative emotional disorders [12, 13]. In keeping with these findings, inhibiting cytokine production can also alleviate depression-like symptoms [14]. Interestingly, antidepressant drugs targeting 5-hydroxytryptamine (5-HT) can suppress depression-induced microglial activation [15]. Together, these evidences strongly suggest that microglia are involved in the development of PTSD.

Microglial activation is always accompanied by dynamic changes in morphology and polarization [16, 17]. Pathological stimulation can induce morphological alterations of microglia such as extension/retraction of branch processes and changes in soma volume. Although the morphology and expression patterns of immunoregulatory proteins in microglia vary among different brain areas under normal physiological conditions, the morphological alterations seen in the injured brains are region-specific [18, 19]. It has been shown that the number of microglia dynamically changes in a depression model induced by chronic unpredicted stresses [20] and that chronic stress can cause structural reorganization of microglial morphology in the prefrontal cortex [21]. In murine PTSD models, microglial cell hyper-ramification and neuronal dendritic spine loss have been reported [22–24]. However, it is still largely unknown how the microglial status changes in different brain areas. The relationship between the morphology and microglial status under pathological conditions, especially in psychiatric disorders, also remains unclear. The prefrontal cortex (PFC), hippocampus (HP), and amygdala (AMY) have been identified as the major brain areas involved in fear emotion regulation [25–27], and fMRI studies indicate that activity alterations in these regions are involved in the development of PTSD [28, 29]. Therefore, it would be of value to decipher the microglial alterations in these emotion-related brain areas during PTSD development.

Minocycline, a semisynthetic tetracycline with anti-inflammatory and immune-modulatory effects, could attenuate the neuroinflammation and promotes neuroplasticity and neurogenesis. Previous studies have suggested minocycline treatment alleviates the anxiety-like behavior and cognitive impairment [30], and minocycline might be a potential strategy for PTSD therapy in the rat [23, 31]. However, the defining cellular mechanisms of minocycline remain unknown in mice.

Mass cytometry, also known as CyTOF (Cytometry by Time-Of-Flight), is a powerful flow cytometry technique developed in recent years. Similar to regular flow cytometry, mass cytometry can determine protein expression at a single-cell level. However, the advantage of mass cytometry is its supreme sensitivity, achieved by using stable and unique heavy metal isotope-labeled antibodies to recognize cellular markers and analyzing them with a time-of-flight mass spectrometer. Using this technique, the immune cells of the murine CNS have been characterized with high dimensional resolution [32], allowing the description of immune landscapes of the murine brain in steady-state [33], aging and neurodegeneration disease [34], and neuroinflammatory disease [35]. Therefore, CyTOF would be a useful tool to characterize the distribution of immune cells during the development of psychiatric disorders such as PTSD.

In this study, we utilized CyTOF to characterize the immunocytes in PTSD model brains and found that microglia constitute the majority of brain immunocytes in response to stress. We further defined the features of microglia, including number, morphology, and gene expression, in the different brain regions during PTSD development. We found significant correlations among the changes in microglial morphology, gene expression levels of proinflammatory cytokines, and behavioral performance. Importantly, we examined the effect of genetic/pharmacological deletion of microglia or minocycline-mediated microglial suppression on the microglial morphology, gene expression, and behavior in PTSD model mice. Based on our findings, we argue that microglial inhibition offers a therapeutic avenue for PTSD treatment.

## Materials and methods

### Animal housing

All mice used in this study were housed at room temperature, in a 12-h dark/light cycle (8:00 a.m.–8:00 p.m.). Mice had free access to standard rodent chow and water. In order to avoid differences arising due to age and gender, only 2–4-month-old male mice were used in this study. Mice in the same group were housed together, with 3–4 mice in each cage. *Cx3cr1-GFP* mice were used as heterozygous mice. *Cx3cr1*^*creER*^ and *iDTR* mice [36] were purchased from Jackson Laboratory. *Cx3cr1-GFP* mice were a kind gift from Dr. Junwei Hao of Tianjin Medical University. *Thy1-GFP* M line transgene mice were used for spine density analysis.

All animal experiments were approved by the Institutional Animal Care and Use Committee at the Beijing Institute of Basic Medical Sciences (Beijing, China).

### Chemical administration

Sertraline (Cat. S6319, Sigma-Aldrich, Germany) was administered by intragastric gavage (i.g.) at a concentration of 15 mg/kg. Tamoxifen (TAM; Cat. S1238, Selleck, USA) was dissolved in sunflower beads oil containing 5% ethanol. A 10-mg tamoxifen was administered once a day for three consecutive days. For microglia depletion, 1-ug diphtheria toxin (DT; Cat. D0564, Sigma-Aldrich, Germany) was injected intraperitoneally 3 weeks after TAM administration, once a day for three consecutive days. PLX3397 (Cat. S7818, Selleck, USA) was added to the diet at a concentration of 290 mg/kg and fed to the mice 21 days before delivering foot-shocks. Minocycline (Cat. S4226, Selleck, USA) was administered intragastrically at 40 mg/kg/day, 3 days before delivering foot-shocks. Sertraline, PLX3397, and minocycline were continually administered until all the behavior tests were completed.

### Behavior tests

All behavior tests were started at 10:00 a.m. and finished before 5:00 p.m. Each group contained eight or more mice, and their littermates were used as the control groups. All behavior tests were analyzed in a double-blind manner.

### Open-field test

Open-field test was conducted inside a clear box (50 cm × 50 cm × 20 cm). Activity was automatically monitored by ANY-maze software (Global Biotech, USA). The apparatus was washed with a 75% ethanol solution before each mouse was introduced. Each mouse was recorded for 5 min and the total distance, average speed, time speed, and distance traveled in the center area (25 cm × 25 cm) were the parameters that were analyzed.

### Elevated plus maze (EPM) test

The maze consisted of two open arms (35 cm × 5 cm) and two enclosed arms (35 cm × 5 cm × 15 cm) connected to a common central platform (5 cm × 5 cm). The apparatus was raised to a height of 50 cm from the floor and was lit by a dim light placed above the central platform. The maze was washed with a 75% ethanol solution before each mouse was introduced. Time spent and distance traveled in open arms versus close arms were measured for a period of 5 min.

### Electric foot-shock procedures

The procedure for electric foot-shocks was adapted from Zhang et al. [37] and Qiu et al. [38]. Electric foot-shocks were carried out in a fear-conditioning chamber (35 cm × 20 cm × 20 cm) (Jiliang Tech, China). After a 5-min adaptation period, 15 intermittent, inescapable foot-shocks were delivered to the mice (15 times, intensity: 0.8 mA; interval: 10 s; duration: 10 s). The control group mice were placed in the same chambers without stimulation for a total of 10 min to adapt to the same circumstance. Repeat stimulation on the second day enhances fear memory and increases the chances of developing PTSD. A 75% ethanol solution was used to wipe the chamber before each mouse was introduced, to avoid any effects of feces and odor.

### Contextual freezing measurement

In the fear contextual test, mice who have previously experienced foot-shocks will freeze intermittently when exposed to the chamber where the foot-shocks were delivered. This freezing behavior is associated with context-induced fear memory [39]. All mice were tested three times at different time points, with each test lasting for 5 min. The total cumulative freezing time and percentage of time spent frozen were recorded and analyzed by DigBehv software (Jiliang Tech, China).

### Immunohistochemistry and immunofluorescence

Immunohistochemistry was performed as previously described [8]. In brief, the mouse brains were fixed with 4% paraformaldehyde after perfusion with saline. The fixed brains were dehydrated in 30% sucrose (in PBS). The 20-μm-thick coronal sections were cut throughout the whole brain, and the sections were washed with PBS and incubated with 3% hydrogen peroxide (H_2_O_2_) for 15 min to inhibit endogenous peroxidases. After three washes with PBS, the sections were blocked for 1.5 h with blocking buffer (0.3% Triton X-100 + 10% goat serum in PBS) at room temperature, followed by incubating with rabbit monoclonal anti-IBA1 (1:600; Cat. 019-19741, WAKO, Japan) and then visualized with biotinylated goat anti-rabbit IgG (1:200, Vectastain ABC kit, Vector Laboratories, USA), followed by streptavidin-conjugated horseradish peroxidase (Vectastain ABC kit, Vector Laboratories, USA) staining. The primary and secondary antibodies were diluted in a blocking buffer. For immunohistochemistry, positive immunostaining was visualized with 3,30-diaminobenzidine (DAB kit, Zhongshanjinqiao, China). Stained sections were mounted onto slides and imaged using Nanozoomer (Hamamatsu, Japan). For immunofluorescence, Alexa Fluor 488-conjugated secondary antibody (1:400, Invitrogen, USA) was used. Nuclear morphology was visualized using Hoechst 33258 (Sigma, USA). *Cx3cr1-GFP* mice and *THY1-GFP* mice were stained only with the Hoechst. Immunofluorescence was imaged using a Nikon A1 confocal microscope (Nikon, USA).

### Image analysis

Images were captured using Nanozoomer (Hamamatsu, Japan) and Nikon A1 confocal microscope (Nikon, USA). The number of microglia and their soma area was automatically measured at ×20 magnification for a defined area by Image Pro Plus (Media Cybernetics, Inc) (import image—count and measure objects—select color—count—view measurement data). For precision, we counted 2–3 fields of the targeted brain area for each slice, 5 consecutive slices from each mouse were analyzed and the average microglial density was calculated.

The skeleton analysis was done using ImageJ software (National Institutes of Health, USA). The images prepared for skeleton analysis were captured as a Z-series stack (20 μm) using Nikon A1 confocal microscope. Z-stack images were condensed into a maximum intensity projection image and converted to 8-bit using an ImageJ plugin and then skeletonized using the Skeletonize (2D/3D) plugin. Microglial process number and length were analyzed using the AnalyzeSkeleton plugin. The resulting parameters were used as measures of microglia morphology.

The spine density analysis was done by NeuronStudio software as described [40]. The images prepared for spine density analysis were capture with a laser scanning confocal microscope using ×40 oil-immersion objective (zoom × 6) at Excitation 488. Z-stack image was merged by ImageJ as above described, and the spine was auto-recognized by NeuronStudio.

### Quantitative real-time PCR

The experiment was performed as previously described [7]. Briefly, total RNA was extracted from the mouse brain tissue using TRIzol (Thermo Fisher, USA). Reverse transcription was performed using random primers. Quantitative PCR was performed using UltraSYBR supermix with ROX (CWBIO, China) and detected by ABI QuantStudio 3 (Thermo Fisher, USA) apparatus. The housekeeping gene ACTB was used as an endogenous control. Gene expression levels were expressed as 2^-ΔCt^. Primer sequences for qPCR are listed in Supplementary Table 2.

### Tissue harvesting and single-cell dissociation

After the first contextual fear response test (day 3), mice were sacrificed, their brains perfused with saline, and the brain parenchyma harvested. Scissors were used to cut the brain parenchyma into small pieces, which were then digested for 30 min at 37 °C in digestion buffer (PBS containing 2% FBS and 2 mg/ml collagenase IV). The samples were homogenized with a syringe and filtered through a 70-mm-cell strainer. After centrifugation at 600 g for 6 min, the acquired pellet was resuspended in 37% Percoll (GE Healthcare, USA) in PBS. This suspension was subjected to gradient centrifugation, with the gradients spanning 70% Percoll in PBS, 30% Percoll in PBS, and only PBS (2000 g for 30 min at 4 °°C). The immunocytes were collected at the 37–70% interphase and washed once in PBS. The samples were then ready for staining with the mass cytometry antibody.

### Mass cytometry

The metal isotope-labeled antibodies used in mass cytometry were made using antibody-labeling kits from Fluidigm (Fluidigm, USA), and all the experiments were performed according to the manufacturer protocols. We first performed tests to ensure that all antibodies were effective and that the parameters were informative. Five different anti-CD45 antibodies conjugated with Pd-104, Pd-105, Pd-106, Pd-108, and Pd-110 were used to label live cells [41]. Then, composite samples were incubated with a cocktail of primary antibodies. Barcoded composite samples were loaded onto a Helios mass cytometer (Fluidigm, USA), and the data were analyzed with MATLAB (MathWorks, China) and Cytobank software (Cytobank, USA). The results were present in the viSNE map, heat map, and Flow Self Organizing Map (FlowSOM).

### Statistical analysis

Statistical analyses were performed using ANOVA followed by Tukey’s post hoc test or by a two-tailed Student’s *t* test, depending on the dataset. All values are expressed as mean ± SEM. *p < 0.05, **p < 0.01, and ***p < 0.001 denote the significance thresholds.

## Results

### Foot-shocks induce mice PTSD-like symptoms

We established a mouse model of post-traumatic stress disorder (PTSD) (Figure S1A) and found that two rounds of foot-shocks significantly increased the contextual fear response on days 3, 8, and 15 as compared to control group mice (Figure S1B-D). Administration of sertraline, a commonly used antidepressant, significantly alleviated this fear response. Consistent with this result, in the open-field test, we found that foot-shock exposure largely reduced the time and distance of locomotion in the center area, and administration of sertraline rescued this phenomenon (Figure S1E-H). Together, these findings indicated that the electric foot-shock model was sufficient to induce PTSD in mice.

### Microglia are the major brain immune cells that respond to PTSD

**Fig. 1:**
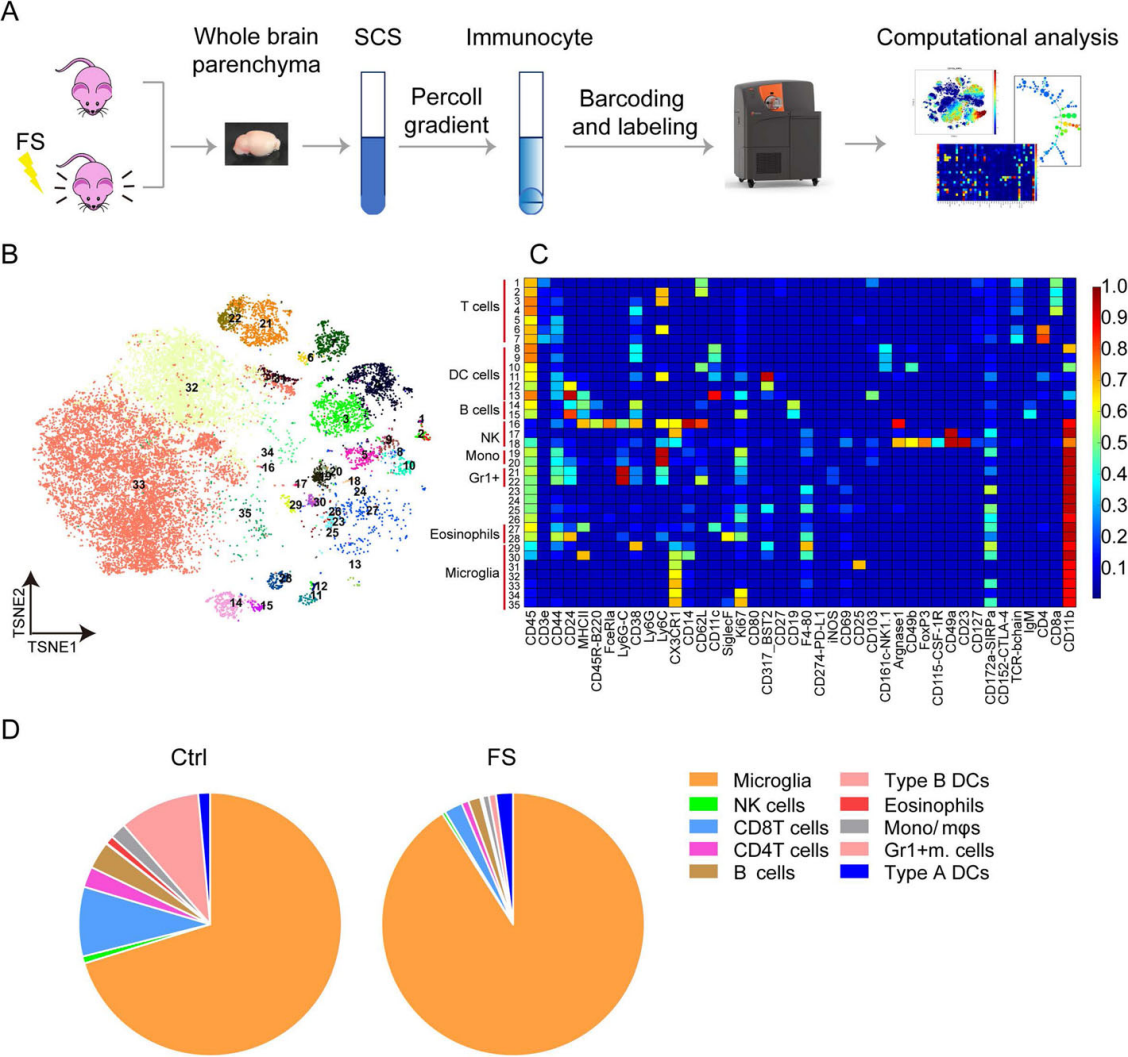
Identification and characterization of immunocyte populations in the mouse brains by mass cytometry. **a** Schematic of mass cytometry procedure. In each group, the brains were separated from five mice, dissociated into single-cell suspensions, mixed as a single sample, barcoded, pooled, and stained with metal-tagged primary antibodies. The samples were analyzed by the CyTOF machine, and immunocyte populations were identified and characterized using visualization tool for statistical epistasis networks (viSNE) and heatmap analysis. **b** viSNE map displaying immune cells from control mice. Colors represent different cell populations clustered by Flow Self Organizing Map (FlowSOM). **c** Clustering and expression level of functional markers in immunocytes from the naïve brains. **d** Frequency distributions of immunocytes in the control and PTSD model brains. (FS foot-shocks, SCS single-cell solution)

In order to study changes in immunocytes from the brains of PTSD mice, we utilized CyTOF technology to dissect immunocyte distributions in control and PTSD mice (Fig. 1a). Five mouse brain samples each from the control or PTSD groups were mixed and labeled with CD45 antibodies conjugated with 42 different isotope-labeled antibodies, including immunocyte-specific and some functional markers (Supplementary Table 1). The immunocytes were categorized into different clusters according to the expression profiles of marker genes (Fig. 1b, c, Figure S2). Consistent with previous reports, multiple immune cell types were identified in the naïve mouse brains, including microglia (CD11b^+^CD45^low^ CX3CR1^+^ F4/80^+^) and various CD45^high^ cells which were mainly from meninges and choroid plexus [33, 42], such as CD4^+^ T cells (CD45^high^TCR-β^+^ CD4^+^), CD8^+^ T cells (CD45^high^TCR-β^+^ CD4^+^), type A dendritic cells (DCs - CD45^high^CD11c^+^CD11b^+^), type B DCs (CD45^high^CD11c^+^CD11b^-^), Gr-1^+^ myeloid cells (CD45^high^CD11b^+^Gr-1^+^), monocytes (CD45^high^CD11b^+^Ly6C^+^), and eosinophils (CD45^high^CD11b^+^CD24^+^ CD44^+^siglec-F^+^). Importantly, we found that more than 70% of these immune cells were microglia (Fig. 1d). Moreover, the immune homeostasis was disrupted in the brains of PTSD mice, characterized by the increased ratio of microglia/immunocytes. This suggested that microglia were the cells that responded to the development of PTSD in mice. Together, these results show that microglia were the dominant immune cell type in the mouse brains and were produced in higher numbers in response to the development of PTSD.

**Fig. 2:**
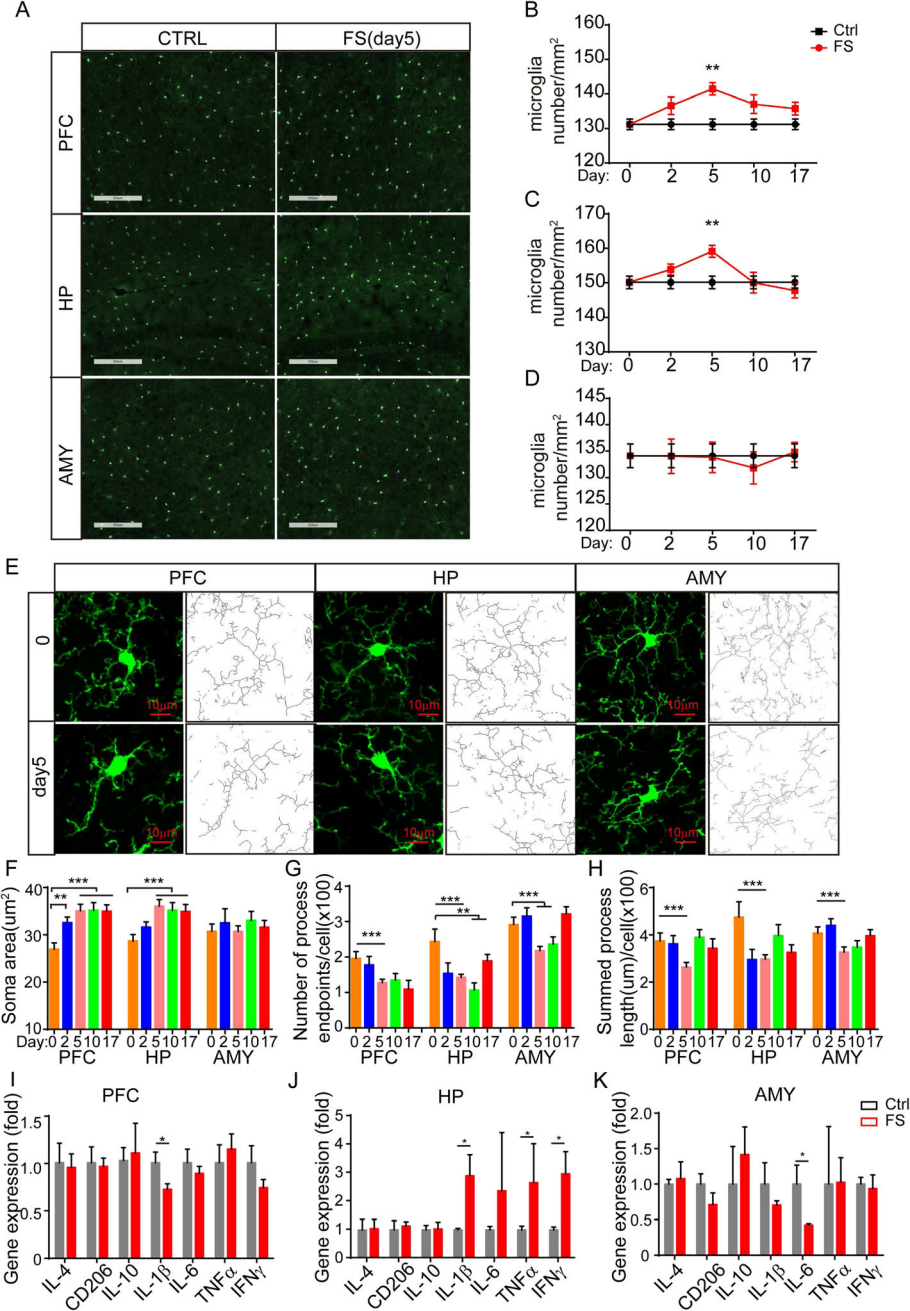
Dynamic alterations of microglial status in PTSD development. **a** Representative picture of microglia density in different brain areas of *Cx3cr1-GFP* transgenic mice of the control and foot-shock groups; scale bar = 200 μm. **b** Microglial density alterations during PTSD development in PFC area (*n* = 3–4 for each group). **c** Microglial density alterations during PTSD development in HP area (*n* = 3–4 for each group). **d** Microglial density alterations during PTSD development in AMY area (*n* = 3–4 for each group). **e** Representative image of microglia morphology and skeletonized inset in different brain areas of *Cx3cr1-GFP* transgenic mice at different time points after foot-shock exposure, scale bar = 10 μm. **f** Statistical analysis of microglial soma area from different brain regions at different time points (*n* = 3–4 mice for each group, 50 cells were analyzed per mouse). **g** Microglial process end points/cell (*n* = 3–4 mice for each group, 50 cells were analyzed per mouse). **h** Microglial process lengths (*n* = 3–4 mice for each group, 50 cells were analyzed per mouse). Data are expressed as mean ± SEM, *p < 0.05, **p < 0.01, ***p < 0.001 for indicated cooperation (ANOVA). **i** Effects of PTSD on the mRNA expression levels of neuroinflammatory genes in PFC, **j** HP, and **k** AMY on day 5; *p < 0.05 (Student’s *t* test). (PFC prefrontal cortex, HP hippocampus, AMY amygdala)

To further define the number and morphology changes in microglia during PTSD development, *Cx3cr1-GFP* mice were utilized. The PFC, HP, and AMY have been reported to be the major brain regions involved in PTSD development; therefore, we examined the changes in microglia from these brain regions at different time points. The number of microglia in the PFC was significantly increased upon delivering foot-shocks, with the highest levels on day 5 and increasing trends on days 10 and 17 (Fig. 2a, b). In the HP, the number of microglia increased on day 5, but quickly recovered on day 10 (Fig. 2c). However, the number of microglia in AMY showed no significant changes (Fig. 2d). These results suggest that microglial numbers are altered in a time- and brain region-dependent manner during the progression of PTSD.

### Dynamic alterations of microglial status throughout PTSD development

Given that microglial activation is always accompanied by dynamic changes in morphology [43], we examined whether microglial morphology was changed in PTSD condition. Foot-shock exposure significantly altered the microglial morphology in the PFC and HP, characterized by enlarged soma area, reduced branch length, and reduced arborization (Fig. 2e–h), especially on day 5 (Fig. 2f–h). However, in AMY, the microglia showed only slight changes. These observations suggest that changes in microglial morphology also occur in a time- and region-specific manner.

Next, we examined the gene expression profiles during PTSD progression. Foot-shock exposure did not change the expression profiles of the PFC and AMY (Fig. 2i–k). However, there were dramatic alterations in the HP, with significant increases of IL-1β, IL-6, TNF-α, and interferon-γ (IFN-γ). Thus, we were able to demonstrate region-specific alterations of microglial gene expression during PTSD progression.

### Foot-shocks reduced the density of the dendritic spines of pyramidal neurons in the CA1 region

As previously reported, hippocampal volume reductions and loss of gray matter in PTSD patients are primarily due to loss of dendrites and their synapses [44]. Pathology studies found synaptic regression was accompanied by microglial activation [22]. We then assessed the density of dendritic spines on pyramidal neurons using *THY1-GFP* M line mice. As the results have shown, foot-shocks decreased the density of the dendritic spine at day 5 after foot-shocks in CA1 regions, especially mushroom and stubby type (Figure S3A and B).

### Genetic/pharmacological depletion of microglia alleviated PTSD-like symptoms in mice

**Fig. 3:**
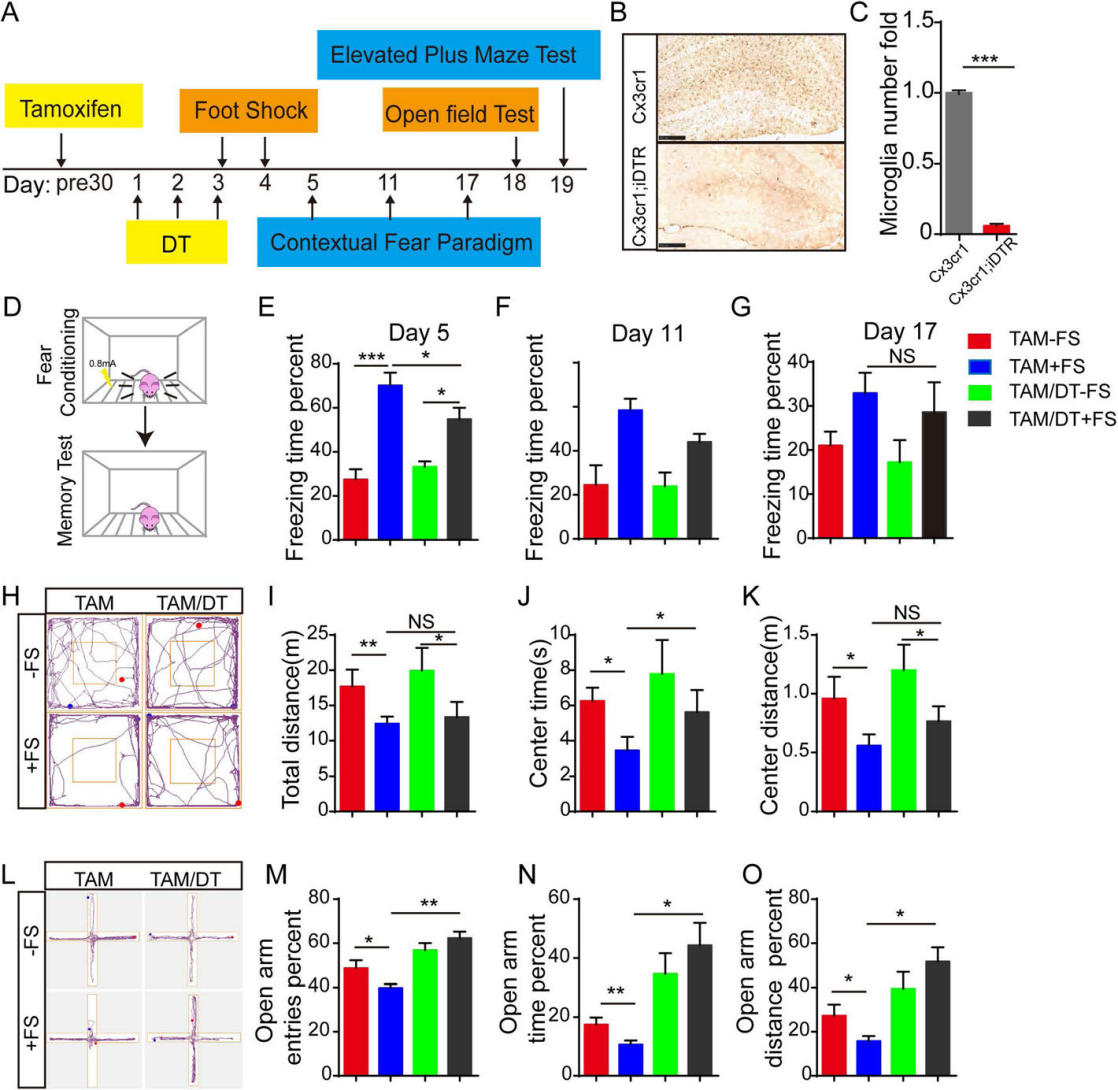
Microglial depletion alleviates PTSD symptoms. **a** Schematic representation of experimental procedure. Tamoxifen was administrated 30 days before DT injection. DT was intraperitoneally injected on −TAM + DT + FS mice, and saline was injected as a control on –TAM − DT + FS mice. At days 3 and 4, foot-shocks were delivered to mice. At days 5, 11, and 17, contextual fear recall test was performed. At day 18, open-field test was performed. At day 19, elevated plus maze test was performed. **b** IHC staining shows microglia depletion efficiency after DT injection 3 days. **c** Statistic data shows microglia number decreased after DT injection. ***p < 0.001 (Student’s *t* test). **d** Representative foot-shock delivery and contextual fear freezing test methods. **e**–**g** Effect of microglial deletion on foot-shocks induced contextual fear freezing test. **h** Representative image of mouse track plots in open-field test. **i**–**k** Statistical analysis of mouse performance in open-field test. **l** Representative image of mouse track plots in EPM test. **m**–**o** Statistical analysis of mouse performance in EPM test; *n* = 10, data are expressed as means ± SEM, *p < 0.05, **p < 0.01, ***p < 0.001 (ANOVA). (DT diphtheria toxin, TAM tamoxifen, NS no significant)

The depletion of microglia in the murine brains has been effectively performed in multiple previous studies, providing an effective way to study the role of microglia in vivo [36, 45]. We used *Cx3cr1*^*creER*^*;iDTR* mice administered with tamoxifen to express the diphtheria toxin receptor (DTR) specifically in microglia. We then administered diphtheria toxin (DT) to deplete microglia in the central nervous system while leaving other CX3CR1^+^ populations intact (Fig. 3a). To examine the efficiency of microglial deletion, brain slices were stained by IBA1 antibody 3 days after the first DT administration and saw that about 90% of microglia in the brain had been removed (Fig. 3b, c). We found that in the PTSD model microglial deletion group mice significantly reduced the freezing time in the contextual recall test as compared to the control group (Fig. 3d–g). In the open-field test, the PTSD model microglial deletion group mice increased the time spent and the distance traveled in the central zone (Fig. 3h–k). Meanwhile, in the EPM test, the PTSD model microglial deletion group mice increased the percentage of open arm entries, the percentage of time spent in open arms, and the percentage of open arm distance traveled (Fig. 3l–o). Collectively, these results suggest that microglial deletion after foot-shock delivery alleviates PTSD-related behaviors in mice.

It has been reported that a genetically ablated microglial population can be restored within 2 weeks of deletion [46, 47]. In our research, we found that microglia repopulated within 14 days after DT treatment; therefore, the microglial number was actually restored when we performed the behavior tests (open-field test and elevated plus maze test) (Figure S4A and S4B). This raises the question of whether the observed improvement in PTSD symptoms is due to deletion or repopulation of microglia. To address this question, a chemical compound, PLX3397, which has been reported to effectively delete microglia, was orally administrated to mice for 3 weeks [48]. The mice then received foot-shocks and underwent behavior tests. The compound PLX3397 was continually administered until all the behavior tests were completed, to sustain the deletion of microglia (Figure S4C and S4D). We found that deletion of microglia by the administration of PLX3397 significantly decreased the contextual fear freezing time (Figure S4E-G), increased the time spent and the distance traveled in the central zone (Figure S4H-K), and slightly improved the activity in open arms in the EPM test (Figure S4L-S4O). These results reveal that microglial depletion alleviates PTSD-like behaviors in mice, implicating microglial activation in the development of PTSD-like phenotypes.

**Fig. 4:**
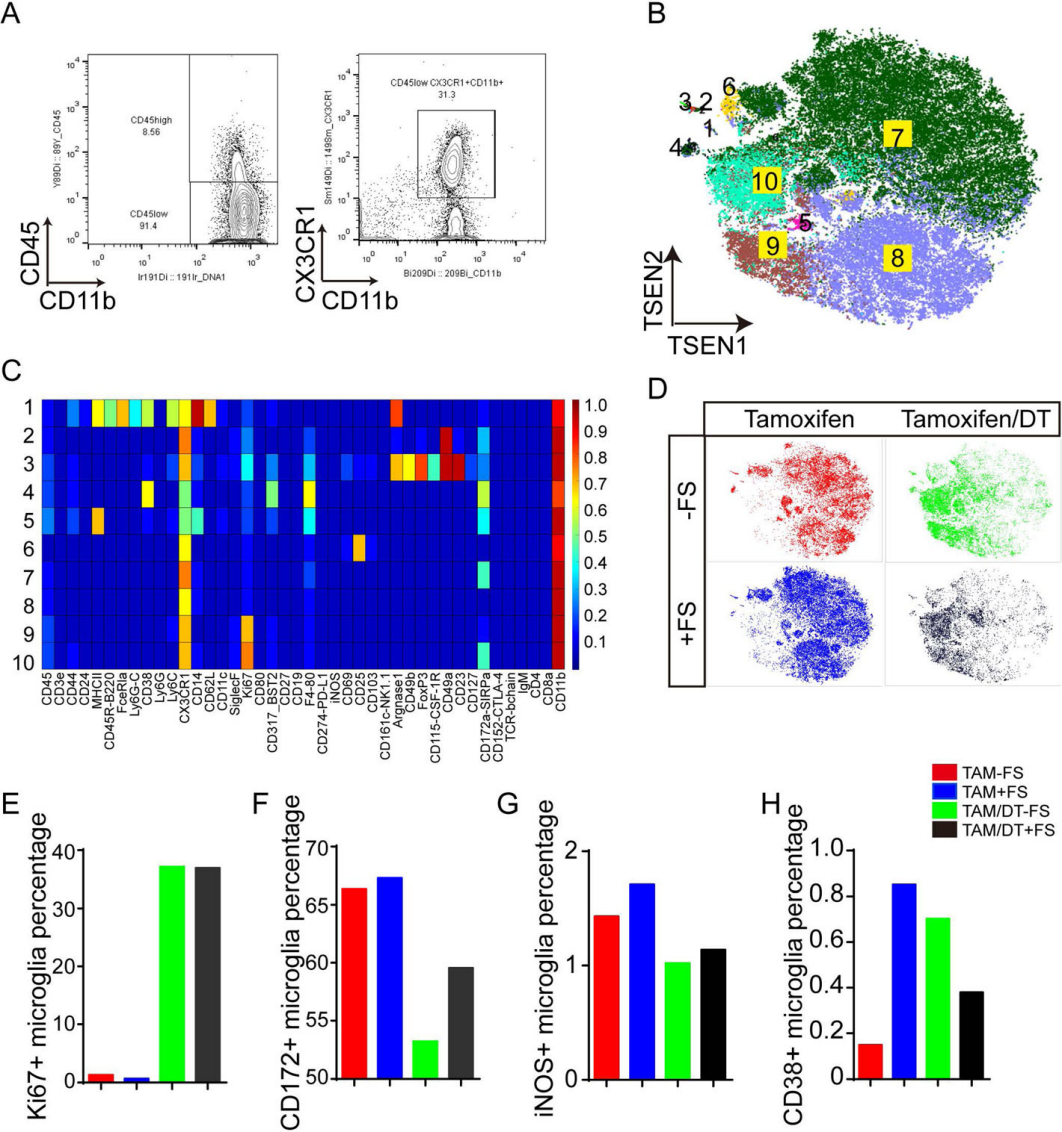
Microglial depletion alleviates PTSD-like symptoms by changing the whole microglial status. **a** Microglia were gated as CD11b^+^CD45^low^CX3CR1^+^ cells. **b** viSNE map displaying microglia subtypes from control mice. Colors represent different cell populations clustered by Flow Self Organizing Map (FlowSOM). **c** Mean expression of mass cytometry panel markers on each microglia subset. **d** viSNE map displaying microglia landscape from each group. **e** Ki67^+^ microglia, **f** CD172^+^ microglia, **g** iNOS^+^ microglia, and **h** CD38^+^ microglia/total microglia percentage in each group

In order to further reveal the mechanism of microglia deletion alleviates PTSD symptoms, we performed mass cytometry experiments and examined the alteration of microglial status among wildtype group mice, foot-shock group mice, microglial deletion group mice, and microglial deletion with foot-shock group mice. Microglial subtypes were gated as Cx3cr1^+^CD11b^+^CD45^low^ population (Fig. 4a), and microglial subtypes were further categorized by FlowSOM-guided clustering according to the expression profiles of marker genes and showed in a t-SNE map (Fig. 4b, c). Microglial deletion significantly altered the microglial patterns (Fig. 4d). Similar to the result that has been reported previously [47], the ratio of Ki67^+^ microglia was markedly increased in the microglial deletion groups (Fig. 4e), indicating that the repopulated microglia were due to the high proliferation. We further analyzed the expression levels of functional markers, including CD172, iNOS, and CD38, which were increased by foot-shock treatment (Fig. 4f–h). Interestingly, microglia deletion significantly downregulates the gene expression under foot-shocks (black bar vs blue bar in Fig. 4f–h). Together, these results indicate that microglial depletion alleviates PTSD-like symptoms by reducing microglia-associated inflammation.

### Inhibition of microglial activation by minocycline alleviates PTSD-like symptoms

**Fig. 5:**
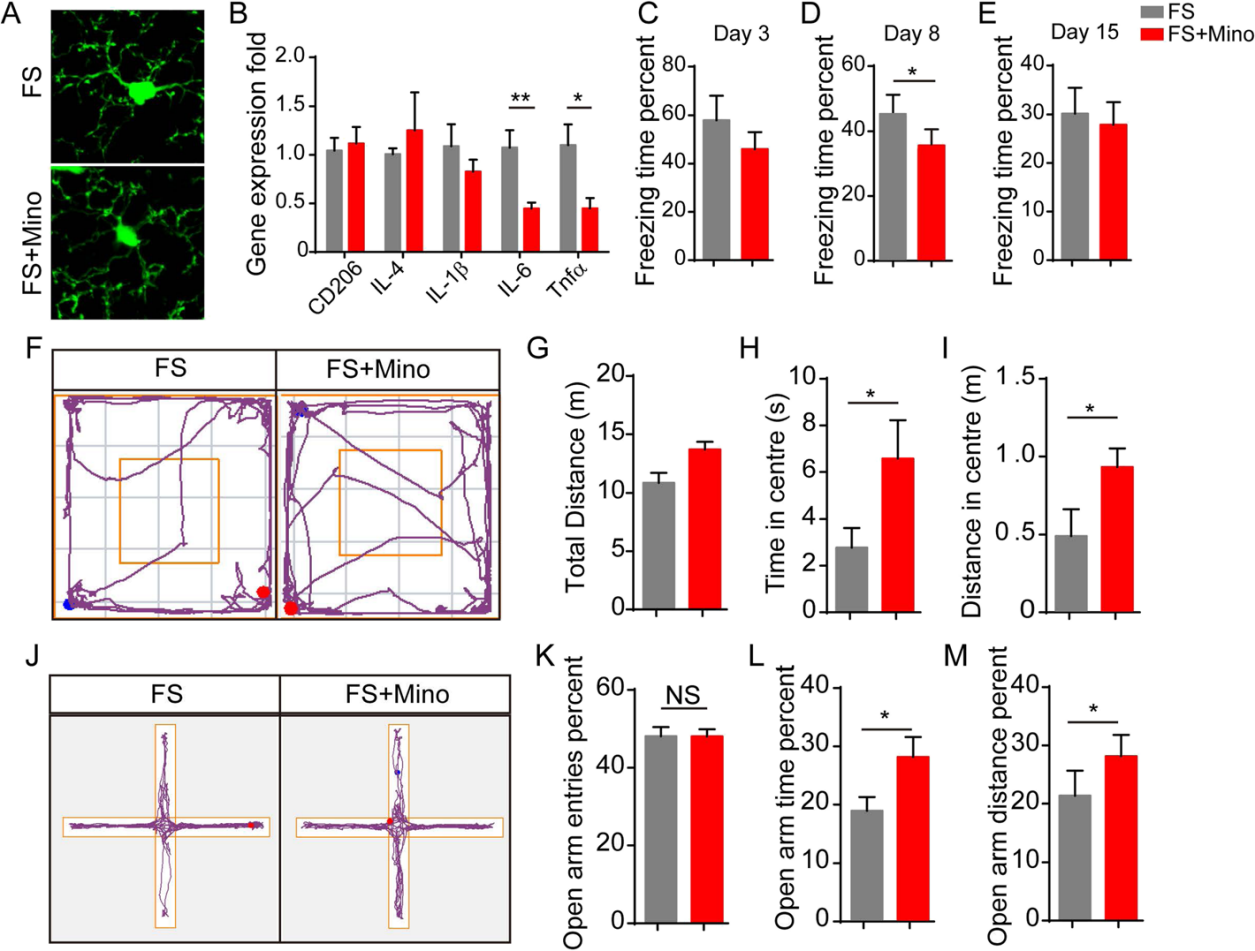
Minocycline treatment alleviates PTSD-like phenotype by suppressing microglial activation. **a** Microglial morphology in the HP changes upon minocycline treatment. **b** Microglia-derived inflammatory gene expression in HP after minocycline treatment, *p < 0.05 (Student’s *t* test). **c–d** Effect of minocycline on PTSD mice in fear freezing test. **f–i** Effect of minocycline on PTSD mice in open-field test. **j–m** Effect of minocycline on PTSD mice in EPM test; *n* = 12, data are expressed as means ± SEM, *p < 0.05, **p < 0.01, ***p < 0.001 (Student’s *t* test). (Mino minocycline, NS no significant)

To further illustrate the role of microglial activation and neuroinflammation, we examined the effect of minocycline, a drug that suppresses microglial activation, on PTSD symptoms [21]. We analyzed the changes in microglial morphology and gene expression profiles in the HP. Treatment with minocycline suppressed the microglial activation induced by foot-shock exposure, characterized by increased branch number and branch length, and decreased expression of pro-inflammatory cytokines like IL-1β, IL-6, and TNF-α (Fig. 5a, b). Furthermore, the behavior tests showed that minocycline administration decreased fear contextual response (Fig. 5c–e), improved performance in the open-field test (Fig. 5f–i), and increased activity in open arms in the EPM test (Fig. 5j–m). Additionally, in order to model PTSD properly, the long-term effect of foot-shocks and minocycline treatment has been investigated (Figure S5A). The contextual freezing test showed that mice freezing to the foot-shock context after foot-shock delivery for 30 days (Figure S5B and S5C). The mice exhibited PTSD-like symptoms reflected by decreased total distance and distance in the center area in open-field test and decreased open arm activity in elevated plus maze test (Figure S5B-K). Minocycline treatment attenuated foot-shock mice fear response in contextual freezing test and alleviated anxiety behaviors in open-field test and EPM test (Figure S5B-K). Overall, these results suggest that suppression of microglial activation can alleviate PTSD-like behaviors induced by foot-shock exposure.

## Discussion

**Fig. 6:**
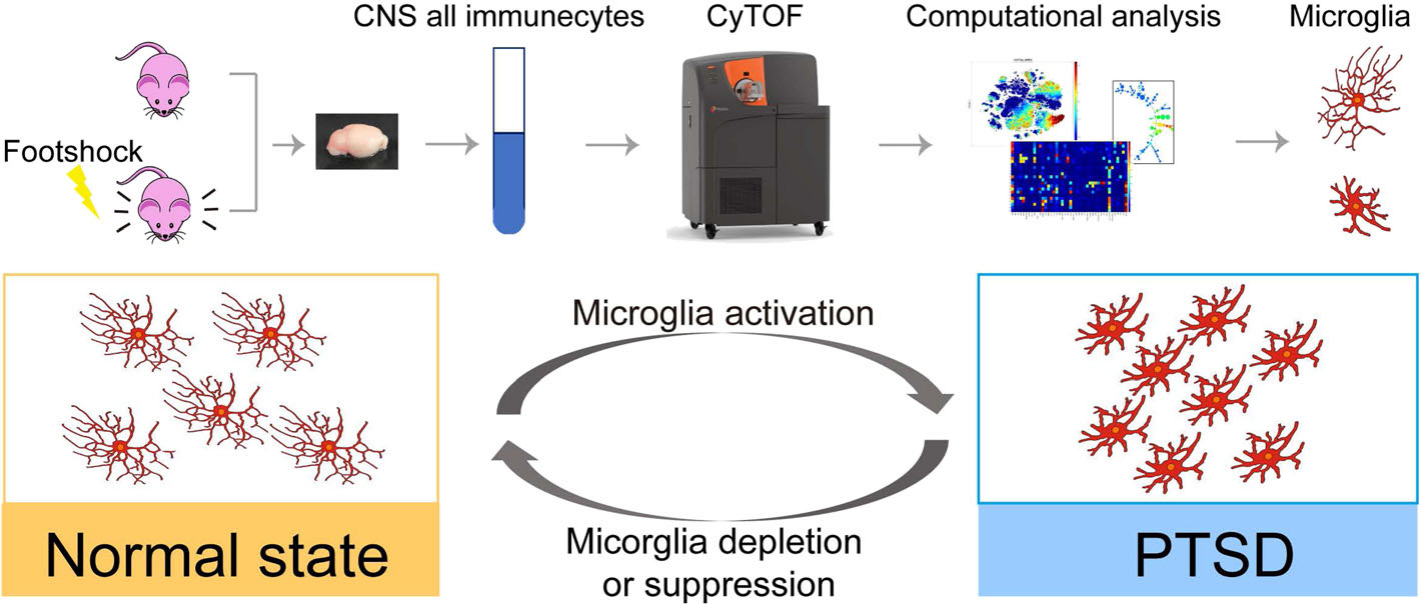
Graphic abstract of microglia activation in PTSD and microglia deletion and suppression alleviates PTSD symptoms

Previous studies on the development of PTSD mainly focused on abnormalities of neuronal function, such as dysregulation of neural circuits and damaged brain structures [49, 50]. Thus, the involvement and role of non-neuronal cells during PTSD remains largely undefined. In this study, we found that PTSD development alters the activation of microglia in certain brain regions. This temporal and spatial alteration of microglial cells during the development of PTSD provides a direct link between microglial activation and mental disorders. Importantly, microglial depletion or inhibition alleviated PTSD-like behaviors, implying that targeting microglia to counter neuroinflammation offers a potential therapeutic avenue for PTSD (Fig. 6).

Currently, there is no standard murine model of PTSD, even some putative models could mimic one or more symptoms of PTSD, among which electronic foot-shocks is extensively used in this field. Some of the PTSD mouse models were made by using high-intensity currents (1.0–3.0 mA), such as 1 mA × 2 s × 4 times (6), or 1.5 mA × 2 s × 2 times (7), or single 1.5 mA × 2 s (8). In our study, we used 0.8 mA × 10s × 15 times per day. Compared with the higher intensity currents (1.0–3.0 mA), we used 0.8 mA but with a longer duration. Furthermore, repeated foot-shock procedures and exposure to the foot-shock contexts were effective strategies to exacerbate freezing responsiveness and anxiety-like symptom development [51]. Importantly, the electronic foot-shock model can be integrated with other behavior tests for extended study on PTSD symptoms [51, 52]. PTSD-related behavior analysis and dendritic spine density assay showed a successful post-traumatic stress disorder mouse model. Importantly, the model was further proved by using sertraline treatment. Accordingly, an electronic foot-shock model has been used in our study.

In this study, we used only age-matched male mice for the experiments. Previous reports showed that there is a gender difference in mice under foot-shock stress treatment [53, 54]. The reason that the female mice are not usually used might be the anti-inflammatory activity of estrogen [55]. Therefore, the majority of psychiatry- and neurology-related studies, the male mice were generally used [20, 56].

Mass cytometry is a new method in a single-cell resolution that offers a broad, high-dimensional perspective of the immunological milieu in the brain. Our findings with the help of mass cytometry were consistent with previous report that the percentage of microglia was 70–80% in the naïve brain [33]. In Dunja’s study, they identify different subsets of myeloid cells and the phenotypic changes in CNS immune cells during aging and central nervous disease condition [34]. Our work found that in PTSD condition, microglia were globally affected, while only a subset of microglia was significantly altered in neurodegenerative disease. Besides microglia response to chronic stress, we also found other immunocytes changed in this process. Changed DCs, CD4+, and CD8+ T cells may play a role in PTSD pathogenesis. As reported before, CD4+-derived xanthine acts on the oligodendrocytes and triggers the onset of anxiety. Dendritic cells involved in major depression pathogenesis through remodeling of D1 neurons by RhoA/Rho-kinase [57, 58].

The finding that short-term stress exposure results in hippocampal microglial activation was consistent with previous report that acute-electronic-shock results in microglia proliferation and activation [22, 23, 59]. Compared to those single-point or single-brain area studies, the present study performed a systemic analysis of microglial changes at several time points across the major brain areas involved in fear emotion regulation in the acute phase after FS and found the temporal and spatial alterations of microglia status in PTSD development and progress. It has been reported that the number of microglia in HP increases due to proliferation and activation in the acute phase of major depression and drops due to apoptosis in the latter stages of major depression [20]. However, in our model, we failed to see microglial apoptosis, which could be attributed to the short duration of stress exposure. Our results that microglia increase after foot-shocks were consistent with other short-term studies [22]. Microglial morphology changes have also been demonstrated in ischemic stroke and reperfusion processes [60], but parallel analyses of gene expression were not performed in these studies. Recently, several studies have demonstrated the spatial and temporal heterogeneity of microglia [18, 61, 62], pointing to a functional relevance of diversity in microglial morphology in the brain. In our study, we present an analysis of microglia morphology across several brain regions throughout the development of PTSD, and we found dynamic alterations and brain area heterogeneity of microglia in PTSD pathology. Accordingly, microglia gene expression profiles exhibited brain region-specific changes. The neuroinflammation-related gene expression in the hippocampal area was significantly upregulated after PTSD. However, in PFC and AMY, the levels of IL-1β and IL-6 were significantly downregulated, respectively. This discrepancy maybe due to spatial and temporal microglia activation in different brain areas after PTSD treatment, which needs further investigation in the future. The present study confirms specific temporal and spatial activation of microglia in affected brain regions during PTSD progression, which may be underpinned by different gene expression patterns.

The finding that DTR/DT-mediated microglial depletion significantly decreased contextual fear memory and alleviated anxiety-like behavior was consistent with previous study that microglia depletion results in learning and memory deficits [36]. Depletion of microglia is a useful method to study microglial functions in vivo. In contrast to the herpes simplex virus thymidine kinase/ganciclovir (HSVTK/GCV) system, the DTR/DT system specifically deletes microglia without inflicting damage upon the blood-brain barrier [63]. In this study, it is a benefit for PTSD treatment, but this strategy cannot be adopted for human patients. However, minocycline might provide an alternative treatment option for PTSD patients, since we found that minocycline effectively attenuates PTSD behaviors in the acute phase. The effect of minocycline on PTSD pathogenesis corroborates the results of previous studies demonstrating the same phenomenon using the acute major depression model and Alzheimer’s disease model [20, 64].

In our work, the genetic deletion (DTR/DT) and pharmacological deletion (PLX3997) were utilized for microglia deletion, both alleviate PTSD behaviors in mice. Recently, several studies have reported microglia repopulation has the benefit effect following brain injury or reverses brain function deficits in aged mice [65, 66]. In our hands, we also observed that DTR/DTs show a more significant improvement than PLX3997, indicating along with the microglial deletion-mediated neuroinflammation inhibition, and microglia repopulation probably also contribute to PTSD alleviation via some unappreciated mechanism.

Microglia are the primary cellular responders to stress, releasing various inflammatory cytokines upon stress exposure. In accordance with our observations, it has also been reported that neural inflammation accompanies major depressive disorder [67–69]. However, it is still puzzling how microglial activation and neural inflammation alter neuronal functions. It has been proposed that IL-1β, IL-6, and TNFα can directly modulate neuronal plasticity and cause mood disorders [70–72]. Another possibility is that the increased microglia number could enhance synaptic trimming and it is known that synapse loss underpins mood disorders [73]. Importantly, microglia-driven microenvironments are critical for neurogenesis and neuronal function, and hence, it is possible that stress-induced microglial activation might lead to the development of PTSD.

Minocycline has been showing as anti-inflammation chemical, and we used it to inhibit the microglia-mediated activation in mice. We found minocycline effectively attenuated microglia activation and alleviated PTSD symptoms. It has been reported that minocycline has a moderate effect on neurons in vitro [9], and minocycline could have some effect on neuronal plasticity [10]. Here, we treated control mice with the minocycline and we found no obvious behavioral abnormality. It has been reported that hippocampal microglia activation underlies the shared neurobiological mechanism of comorbid PTSD and chronic pain by using minocycline treatment [24]. Inhibition of microglial activation maybe a possible strategy for cotreating PTSD and chronic pain. Furthermore, PTSD might contribute to the pathogenesis of the cardiovascular disease and autoimmune disease, as well as neurodegenerative diseases, though the dysregulated immune system. This work provides a good foundation for further study on the relationship between PTSD and other disease comorbidities.

## Conclusion

Our results demonstrated that microglia are the major brain immune cells that respond to PTSD, and microglial activation plays a critical role in PTSD development. Microglial inhibition is a potential therapeutic strategy for the clinical treatment of PTSD.

## Supplementary Information

**Additional file 1: Figure S1.** Mice exposed to foot-shocks developed PTSD-like behavior. (A) Schematic representation of treatment schedules and the order of behavior tests. As shown, foot shocks (FS) were delivered to mice at day 1 and day 2, contextual freezing paradigm were measured at day 3, day 8, day 15, and open field (OF) test were performed at day 16. FS: foot-shocks, CFP: contextual freezing paradigm, OF: open field test. (B-D) Foot-shocks exposure increased mice contextual freezing, sertraline administration reduced freezing time percent. (E) Representative image of mice track plot in OF test. (F-H) Statistical analysis of mice performance in OF test. n=9, Data are expressed as means ± SEM. * *p*<0.05, **p<0.01, ***p<0.001 (ANOVA). **Figure S2.** High-dimensional characterization of brain lymphocytes. (A) viSNE plots show the expression pattern of representative markers in all brain lymphocytes. **Figure S3.** Decreased the density of dendritic spines of pyramidal neurons in CA1 region in PTSD mice brain. (A) Representative images of third order dendrites in the CA1 regions of the hippocampus. scale bar = 10 μm (B) Statistic graph displaying spine density of third order dendrites in CA1 region. **Figure S4.** Microglia depletion alleviates foot-shocks induced PTSD-like behaviors. (A) IHC staining shows microglia restored after DT administration. (B) Statistic data shows microglia number restored after DT administration. **p<0.01,***p<0.001 (ANOVA) (C) IHC staining shows microglia depletion efficiency after elevated plus maze test with PLX3397 treatment. (D) Statistic data shows microglia number decreased after elevated plus maze test with PLX3397 treatment. (E-G) PLX3397 administration decreased mice freezing behavior. (H-K) PLX3397 administration increased mice activity in center area in OF test. (L-O) PLX3397 administration increased mice activity in open arm in EPM test. n=8, Data are expressed as means ± SEM. * *p*<0.05, **p<0.01, ***p<0.001 (Student’s *t* test). **Figure S5.** Minocycline treatment alleviates PTSD-like phenotype after long term foot shocks exposure. (A) Schematic representation of experimental procedure. (B-C) Effect of minocycline on PTSD mice in fear freezing test. (D-G) Effect of minocycline on PTSD mice in open field test. (H-K) Effect of minocycline on PTSD mice in EPM test; n = 10, data are expressed as means ± SEM, * *p* < 0.05, ** *p* < 0.01, *** *p* < 0.001 (ANOVA). **Supplementary Table 1.** List of antibody panel used in CytoF. **Supplementary Table 2.** QPCR primer sequence.

## Data Availability

All data generated or analyzed during this study are included in this published article and its supplementary information files.

## Abbreviations

PTSD: Post-traumatic stress disorder
TNFα: Tumor necrosis factor α
IL-6: Interleukin-6
IL-1β: Interleukin-1β
CNS: Central nervous system
5-HT: 5-Hydroxytryptamine
PFC: Prefrontal cortex
HP: Hippocampus
AMY: Amygdala
CyTOF: Cytometry by Time-Of-Flight
EPM: Elevated plus maze
IFN-γ: Interferon-γ
DTR: Diphtheria toxin receptor
