# Supplementary material for: Microglial deletion and inhibition alleviate behavior of post-traumatic stress disorder in mice
Source: J Neuroinflammation. 2021 Jan 5;18:7. doi: 10.1186/s12974-020-02069-9 (PMC7786489; doi:10.1186/s12974-020-02069-9)
Supplement: Supplementary file 1 — Additional file 1: Figure S1. Mice exposed to foot-shocks developed PTSD-like behavior. (A) Schematic representation of treatment schedules and the order of behavior tests. As shown, foot shocks (FS) were delivered to mice at day 1 and day 2, contextual freezing paradigm were measured at day 3, day 8, day 15, and open field (OF) test were performed at day 16. FS: foot-shocks, CFP: contextual freezing paradigm, OF: open field test. (B-D) Foot-shocks exposure increased mice contextual freezing, sertraline administration reduced freezing time percent. (E) Representative image of mice track plot in OF test. (F-H) Statistical analysis of mice performance in OF test. n=9, Data are expressed as means ± SEM. * p<0.05, **p<0.01, ***p<0.001 (ANOVA). Figure S2. High-dimensional characterization of brain lymphocytes. (A) viSNE plots show the expression pattern of representative markers in all brain lymphocytes. Figure S3. Decreased the density of dendritic spines of pyramidal neurons in CA1 region in PTSD mice brain. (A) Representative images of third order dendrites in the CA1 regions of the hippocampus. scale bar = 10 μm (B) Statistic graph displaying spine density of third order dendrites in CA1 region. Figure S4. Microglia depletion alleviates foot-shocks induced PTSD-like behaviors. (A) IHC staining shows microglia restored after DT administration. (B) Statistic data shows microglia number restored after DT administration. **p<0.01,***p<0.001 (ANOVA) (C) IHC staining shows microglia depletion efficiency after elevated plus maze test with PLX3397 treatment. (D) Statistic data shows microglia number decreased after elevated plus maze test with PLX3397 treatment. (E-G) PLX3397 administration decreased mice freezing behavior. (H-K) PLX3397 administration increased mice activity in center area in OF test. (L-O) PLX3397 administration increased mice activity in open arm in EPM test. n=8, Data are expressed as means ± SEM. * p<0.05, **p<0.01, ***p<0.001 (Student’s t test). Figure [file 12974_2020_2069_MOESM1_ESM.pdf]

Figure S1

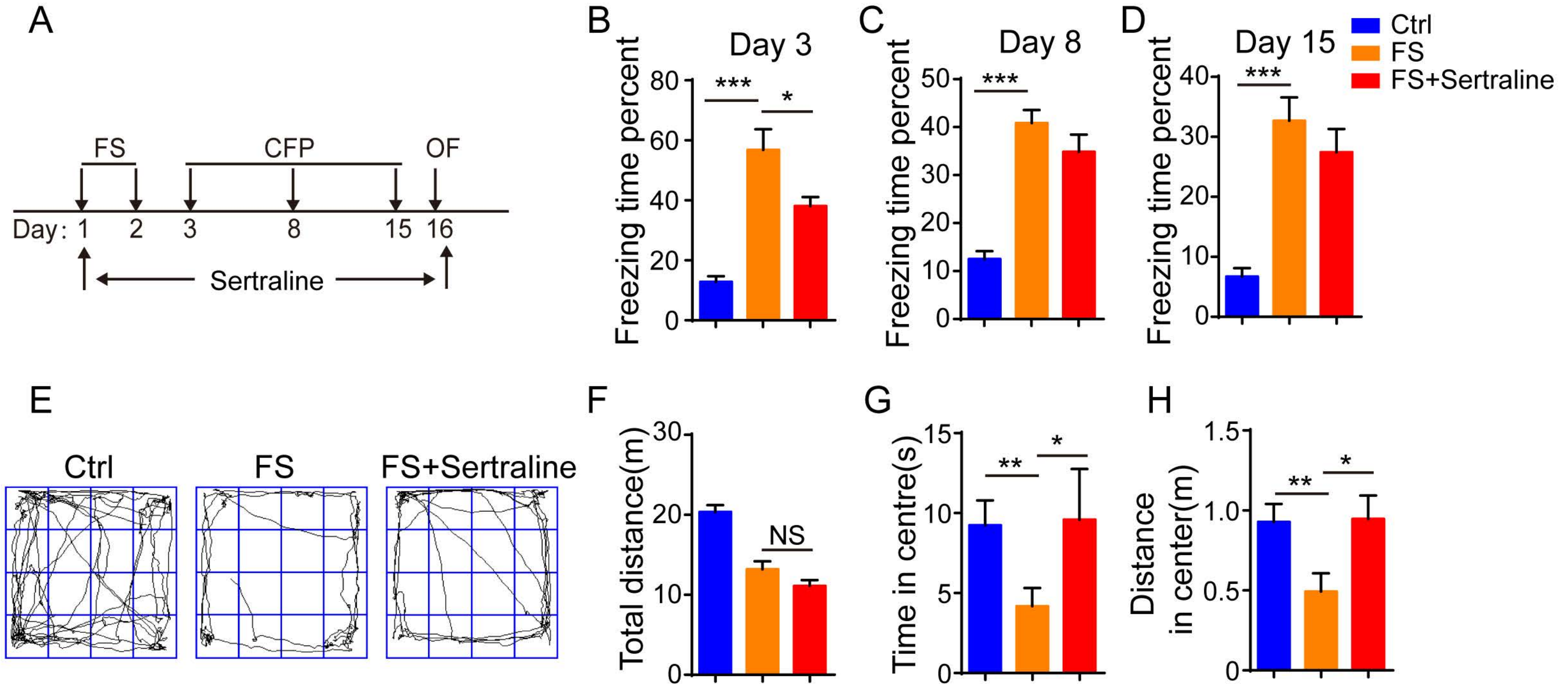
A
FS
CFP
OF
Day: 1 2 3 8 15 16
Sertraline
B
Day 3
Freezing time percent
***
*
C
Day 8
Freezing time percent
***
D
Day 15
Freezing time percent
***
Ctrl
FS
FS+Sertraline
E
Ctrl
FS
FS+Sertraline
F
Total distance(m)
NS
G
Time in centre(s)
**
*
H
Distance in center(m)
**
*

Figure S2

A

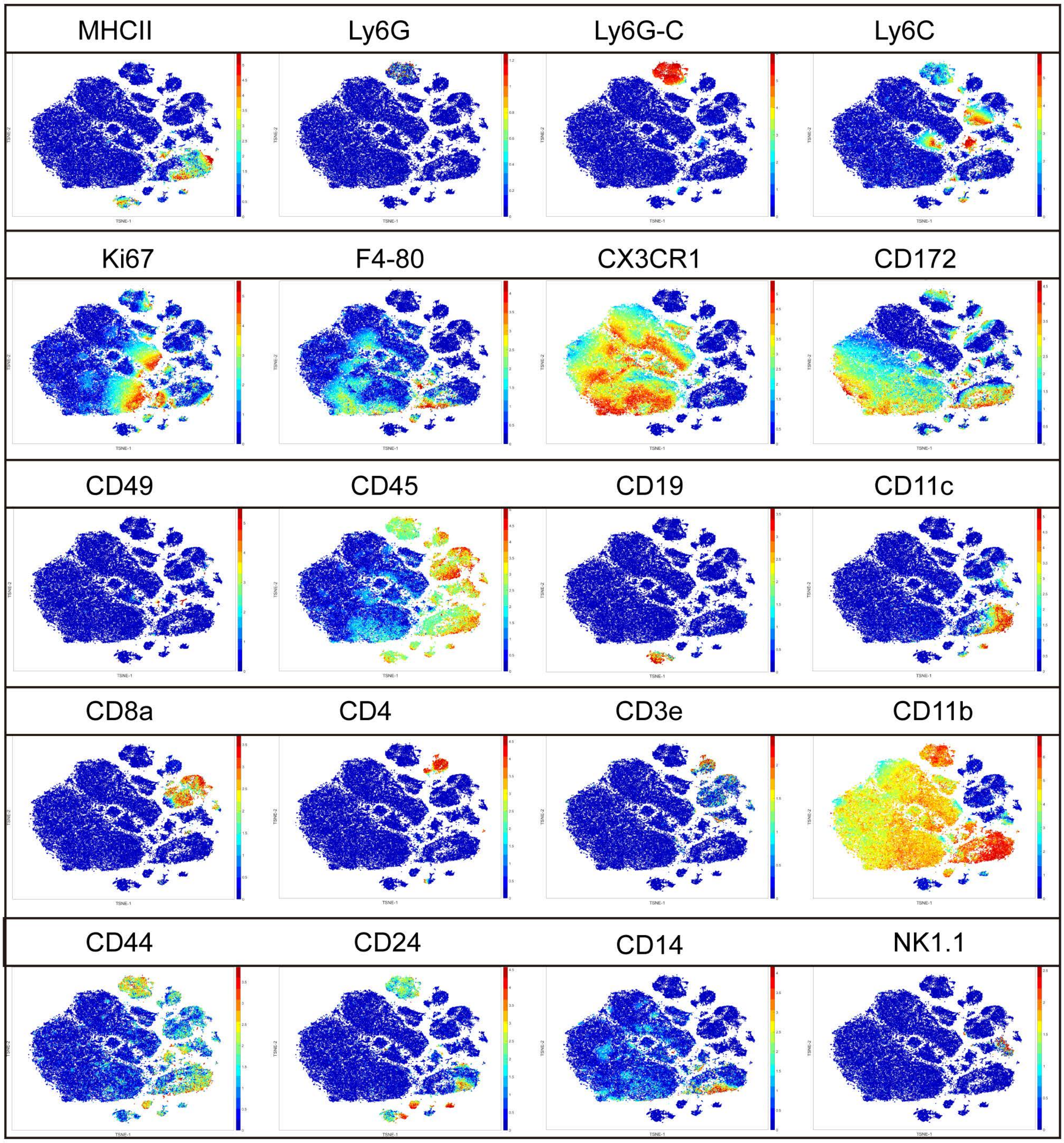
MHCII
Ly6G
Ly6G-C
Ly6C
Ki67
F4-80
CX3CR1
CD172
CD49
CD45
CD19
CD11c
CD8a
CD4
CD3e
CD11b
CD44
CD24
CD14
NK1.1

Figure S3

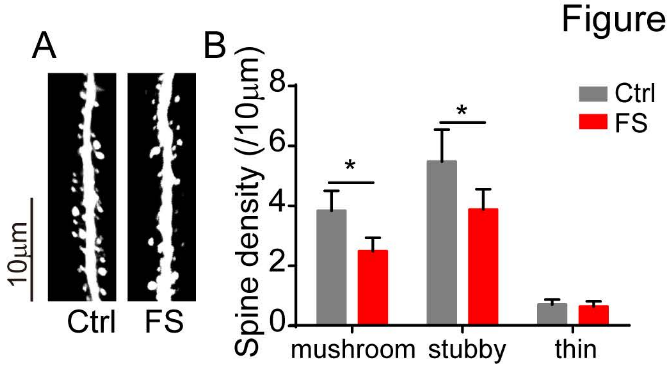
A
B
10μm
Ctrl
FS
Spine density (/10μm)
8
6
4
2
0
*
*
Ctrl
FS
mushroom
stubby
thin

Figure S4

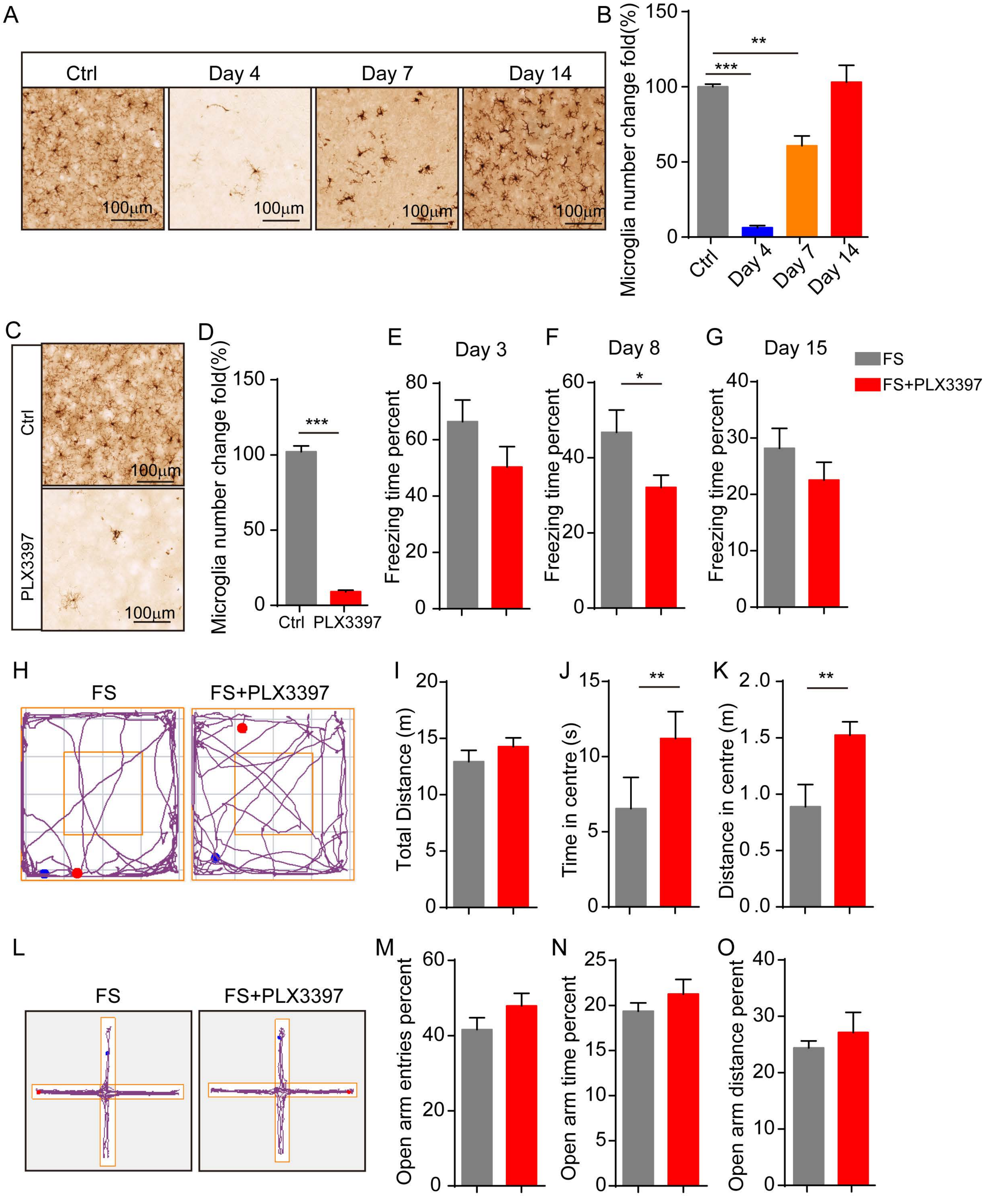
A
Ctrl
Day 4
Day 7
Day 14
100μm
B
Microglia number change fold(%)
***
**
Ctrl
Day 4
Day 7
Day 14
C
Ctrl
PLX3397
D
Microglia number change fold(%)
***
Ctrl
PLX3397
E
Day 3
Freezing time percent
F
Day 8
*
G
Day 15
FS
FS+PLX3397
H
FS
FS+PLX3397
I
Total Distance (m)
J
Time in centre (s)
**
K
Distance in centre (m)
**
L
FS
FS+PLX3397
M
Open arm entries percent
N
Open arm time percent
O
Open arm distance perent

Figure S5

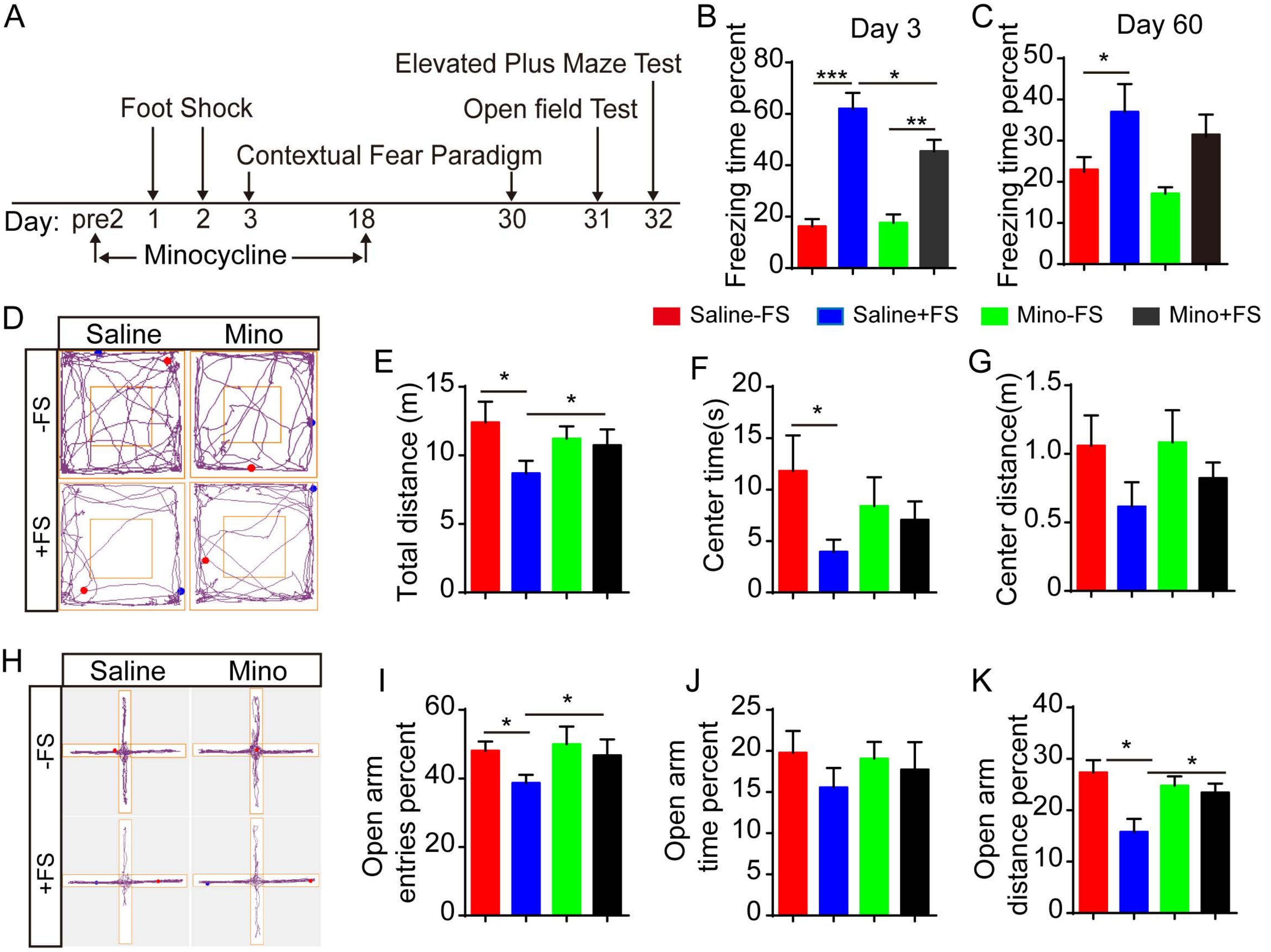

A
Elevated Plus Maze Test
Foot Shock
Open field Test
Contextual Fear Paradigm
Day: pre2 1 2 3 18 30 31 32
Minocycline
B
Day 3
Freezing time percent
C
Day 60
Freezing time percent
Saline-FS
Saline+FS
Mino-FS
Mino+FS
D
Saline
Mino
-FS
+FS
E
Total distance (m)
F
Center time(s)
G
Center distance(m)
H
Saline
Mino
-FS
+FS
I
Open arm entries percent
J
Open arm time percent
K
Open arm distance percent

Figure S1. Mice exposed to foot-shocks developed PTSD-like behavior. (A) Schematic representation of treatment schedules and the order of behavior tests. As shown, foot-shocks (FS) were delivered to mice at day 1 and day 2, contextual freezing paradigm were measured at day 3, day 8, day 15, and open field (OF) test were performed at day 16. FS: foot-shocks, CFP: contextual freezing paradigm, OF: open field test. (B-D) Foot-shocks exposure increased mice contextual freezing, sertraline administration reduced freezing time percent. (E) Representative image of mice track plot in OF test. (F-H) Statistical analysis of mice performance in OF test. $n=9$, Data are expressed as means ± SEM. * $p<0.05$, **$p<0.01$, ***$p<0.001$ (ANOVA).

Figure S2. High-dimensional characterization of brain lymphocytes. (A) viSNE plots show the expression pattern of representative markers in all brain lymphocytes.

Figure S3. Decreased the density of dendritic spines of pyramidal neurons in CA1 region in PTSD mice brain. (A) Representative images of third order dendrites in the CA1 regions of the hippocampus. scale bar = 10 μm (B) Statistic graph displaying spine density of third order dendrites in CA1 region.

Figure S4. Microglia depletion alleviates foot-shocks induced PTSD-like behaviors. (A) IHC staining shows microglia restored after DT administration. (B) Statistic data shows microglia number restored after DT administration. **$p<0.01$,***$p<0.001$ (ANOVA) (C) IHC staining shows microglia depletion efficiency after elevated plus maze test with PLX3397 treatment. (D) Statistic data shows microglia number decreased after elevated plus maze test with PLX3397 treatment. (E-G) PLX3397 administration decreased mice freezing behavior. (H-K) PLX3397 administration increased mice activity in center area in OF test. (L-O) PLX3397 administration increased mice activity in open arm in EPM test. $n=8$, Data are expressed as means ± SEM. * $p<0.05$, **$p<0.01$,***$p<0.001$ (Student's *t* test).

Figure S5. Minocycline treatment alleviates PTSD-like phenotype after long term foot-shocks exposure. (A) Schematic representation of experimental procedure. (B-C) Effect of minocycline on PTSD mice in fear freezing test. (D-G) Effect of minocycline on

PTSD mice in open field test. (H-K) Effect of minocycline on PTSD mice in EPM test; n = 10, data are expressed as means ± SEM, * $p < 0.05$, ** $p < 0.01$, *** $p < 0.001$ (ANOVA).

Supplementary Table1. list of antibody panel used in CytoF

List	mark	List	mark
1	CD45	22	F4/80
2	CD3e	23	CD274(PD-L1)
3	CD44	24	iNOS
4	CD24	25	CD69
5	MHC II	26	CD25
6	B220(CD45R)	27	CD103
7	FcεRIα	28	NK1.1
8	Ly6G/C	29	Argnase I
9	CD38	30	CD49b
10	Ly6G	31	Foxp3
11	Ly6C	32	CD115(CSF-1R)
12	CX3CR1	33	CD49a
13	CD14	34	CD23
14	CD62L	35	CD127
15	CD11c	36	CD172a(SIRPα)
16	SiglecF	37	CD152(CTLA-4)
17	KI67	38	TCR-b chain
18	CD80	39	IgM
19	BST2	40	CD4
20	CD27	41	CD8a
21	CD19	42	CD11b

Supplementary Table2: QPCR primer sequence

Gene symbol	NCBI Gene ID	Forward	Reverse
TNF-α	21926	CAGGCGGTGCCTATGTCTC	CGATCACCCCGAAGTTCAGTAG
IL-6	16193	GCTACCAAACTGGATATAATCAGGA	CCAGGTAGCTATGGTACTCCAGAA
Il-4	16189	GGTCTCAACCCCCAGCTAGT	GCCGATGATCTCTCTCAAGTGAT
Il-10	16153	GCTCTTACTGACTGGCATGAG	CGCAGCTCTAGGAGCATGTG
CD206	17533	CTCTGTTCAGCTATTGGACGC	CGGAATTTCTGGGATTCAGCTTC
β-actin	11461	GGCTGTATTCCCCTCCATCG	CCAGTTGGTAACAATGCCATGT
IFNγ	15978	GATGCATTCATGAGTATTGCCAAGT	GTGGACCACTCGGATGAGCTC
IL-1β	16176	GCAACTGTTCCTGAACTCAACT	ATCTTTTGGGGTCCGTCAACT
